# Paramyxovirus Infections in Ex Vivo Lung Slice Cultures of Different Host Species

**DOI:** 10.3390/mps1020012

**Published:** 2018-03-27

**Authors:** Rory D. de Vries, Linda J. Rennick, W. Paul Duprex, Rik L. de Swart

**Affiliations:** 1 Department of Viroscience, Erasmus MC, P.O. Box 2040, 3000 CA Rotterdam, The Netherlands; r.d.devries@erasmusmc.nl; 2 Department of Microbiology, Boston University School of Medicine, Boston, MA 02118, USA; rennick@bu.edu (L.J.R.); pduprex@bu.edu (W.P.D.)

**Keywords:** lung slice, paramyxovirus, respiratory virus, ex vivo model, infection, pathogenesis

## Abstract

In vivo experiments in animal models of disease are of crucial importance for viral tropism and pathogenesis studies. However, these experiments must be complemented with in vitro and ex vivo experiments. Here, we describe a protocol for the preparation and ex vivo infection of lung slices from different mammalian host species with various respiratory paramyxoviruses expressing fluorescent reporter proteins, and suggest follow-up experiments including immunohistochemistry, flow cytometry and confocal microscopy.

## 1. Introduction

Ex vivo models provide an important bridge between in vitro and in vivo experiments. The use of agarose-inflated lung slices for respiratory virus pathogenesis studies has been described previously [1,2,3,4,5,6]. Here, we describe a protocol in which agarose-inflated lung slices can be kept viable in culture for at least seven days post-necropsy of an experimental animal. The combination of these viable lung slices with recombinant viruses expressing fluorescent reporter proteins [7,8,9] allows for accurate, sensitive and reproducible assessment of respiratory virus infection and dissemination over time. Furthermore, use of these recombinant viruses allows for real time monitoring of infection processes, using multiple methods for measurement of fluorescence (e.g., flow cytometry and confocal laser scanning microscopy). Lung slices are also suitable for analysis by immunohistochemistry, thereby visualizing virus cell tropism and spatial localization of infected cells within the tissue. We have validated this technique by infecting lung slices of multiple host species (cotton rats, ferrets, dogs and macaques) with various paramyxoviruses expressing fluorescent reporter proteins (measles virus (MV), canine distemper virus (CDV), human respiratory syncytial virus (HRSV) and human metapneumovirus (HMPV)) [10]. This technique, however, is directly transferable to different host species and different viruses [11]. 

## 2. Experimental Design

**Figure 1:**
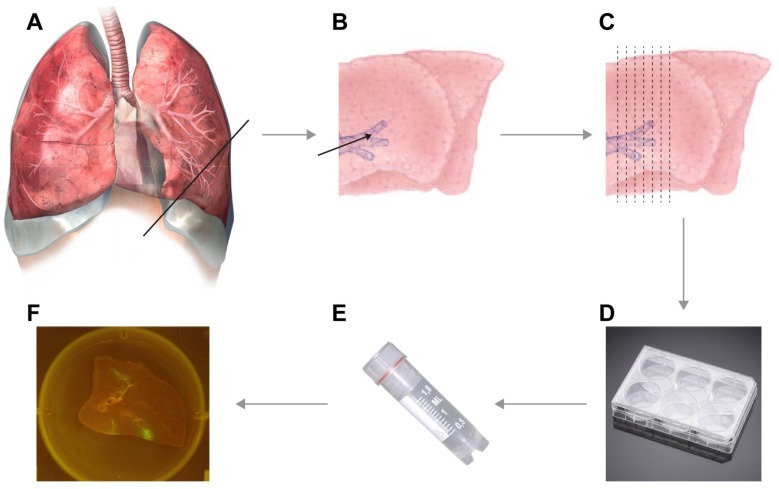
Flow scheme of the experimental design. Lungs are resected during necropsy (**A**) and inflated with warm liquid low-melting point agarose (**B**). After solidification on ice, thin slices of approximately 1-mm thick can be cut by hand (**C**) and are transferred to 6- or 24-well plates pre-filled with culture medium (**D**). Slices can subsequently be inoculated with the desired virus (**E**) and infection of the slices is followed in time (**F**). In this example in panel F, a macaque lung slice infected with recombinant measles virus is shown.

This protocol describes the ex vivo agarose inflation of lungs and generation of slices for virus infection. Lungs should be taken from the experimental animal during necropsy, keeping the material as intact as possible and attached to the primary bronchus and trachea. Using a (blunt-end) needle or flexible catheter, the fresh lungs are inflated through the trachea (or primary bronchus, if inflation of a half lung or single lobe is desired) with low-melting point agarose mixed with culture medium. After solidification on ice, slices can be prepared by hand using microtome blades and cultured in 6- or 24-well plates with culture medium, dependent on the size of the slices. These lung slices are subsequently inoculated with infectious virus (or could be used for any other experimental purpose) and can be followed in time by phase-contrast inverted light microscopy or fluorescence, when reporter viruses expressing fluorescent proteins are used (see Figure 1). In addition, emigrant cells in the culture medium can be harvested in time and analyzed by flow cytometry. Although these emigrant cells could be present due to tissue degradation, they can be detected at early time-points after refreshing the culture medium, suggesting they are mobile emigrant cells. We have detected cells from lymphoid, myeloid and epithelial origin using this method. At the end of the experiment, slices can be used as desired; potential applications include fixation followed by paraffin embedding and immunohistochemistry, or generation of single cell suspensions to be analyzed via flow cytometry.

All animals used in these studies were housed and experiments were conducted in strict compliance with European guidelines (EU directive on animal testing 86/609/EEC) and Dutch legislation (Experiments on Animals Act, 1997). Protocols were approved by the independent animal experimentation ethical review committee DCC in Driebergen, the Netherlands. Animal welfare was observed on a daily basis.

### 2.1. Materials

- Hydroxyethylagarose, Type VII, low gelling temperature (Sigma-Aldrich, St. Louis, MO, USA; Cat. no.: A4018);
- Scissors/forceps/clamp;
- 10 mL syringe (Fisher Scientific, Hampton, NH, USA; Cat. no.: NC9001097);
- Catheter (20G I.V.1 in 25 mm, Patterson Veterinary, Saint Paul, MN, USA; Cat.no.: 32058-46-20);
- Blunt-end needles (Miltenyi Biotec, Bergisch Gladbach, Germany; Cat. no.: 130-091-558);
- Petri dishes, 150 × 15 mm (Fisher Scientific, Hampton, NH, USA; Cat. no.: 08-757-148);
- Cutting board;
- Microtome blade (MX35 Ultra, Thermo Scientific, Waltham, MA, USA);
- Six-, 12-, 24- or 48-wells plates (Greiner, Kremsmunster, Austria);
- Thin-bottom/glass-bottom dishes (e.g., Ibidi, Planegg, Germany; Cat. no.: 81156);
- Fifteen-milliliter tubes (Greiner, Kremsmunster, Austria);
- V-bottom 96-wells plate (Greiner, Kremsmunster, Austria);
- Triton X-100 (Sigma-Aldrich, St. Louis, MO, USA; Cat. no.: T8787);
- 10% buffered formalin (Sigma-Aldrich, St. Louis, MO, USA; Cat. no.: HT501128);
- Hank’s Buffered Saline Solution (HBSS; Lonza, Basel, Switzerland; Cat. no.: BE10-508F);
- Collagenase (Invitrogen, Carlsbad, CA, USA, Cat. no.: 17100-017);
- DNAse (Sigma-Aldrich, St. Louis, MO, USA; Cat. no.: 10104159);
- Cell strainers (100 μm nylon; Becton Dickinson, NJ, USA);
- Red blood cell lysis buffer (Roche, Basel, Switzerland; Cat. no.: 11814389001);
- Dulbecco’s phosphate-buffered saline (DPBS; Invitrogen, Carlsbad, CA, USA; Cat. no.: 14190-250);
- Dulbecco’s modified Eagle’s medium (DMEM; Lonza Bio-Whittaker, Basel, Switserland);
- Ham’s Nutrient Mixture F12 powder (Invitrogen, Carlsbad, CA, USA; Cat. no.: 12500062);
- Glutamax (Invitrogen, Carlsbad, CA, USA; Cat. no.: 35050-061);
- Fetal bovine serum (FBS; Lonza Bio-Whittaker, Basel, Switserland; Cat. no.: 14-501GM);
- Penicillin (100 U/mL)/Streptomycin (100 mg/mL) (Lonza Bio-Whittaker, Basel, Switserland; Cat. No.: DE17-602E);
- Amphotericin (1 μg/mL) (Fungizone, Sigma-Aldrich, St. Louis, MO, USA; A4888);
- Bovine serum albumin (BSA; Sigma, St. Louis, MO, USA; Cat. no.: A1595);
- Ethylenediaminetetraacetic acid (EDTA; Sigma, St. Louis, MO, USA; Cat. no.: ED2SS);
- Paraformaldehyde (PFA; Sigma-Aldrich, St. Louis, MO, USA; Cat. no.: 441244).

### 2.2. Equipment

- Phase-contrast inverted light microscope;
- Macroscopic imaging lamp and emission filter;
- Inverted fluorescence microscope;
- Confocal laser-scanning microscope;
- Flow cytometer.

## 3. Procedure

### 3.1. Inflation of Lungs. Time for Completion: 1 h

1. Prepare low-melting point agarose beforehand.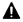 **CRITICAL STEP** Prepare 4% (*w*/*v*) low-melting point agarose by dissolving agarose in phosphate-buffered saline (PBS) and boil for several minutes (e.g., using a microwave oven, be careful for boiling over). As an alternative, agarose can be prepared by autoclaving. Be sure to allow agarose to cool down to 42 °C (e.g., in a water bath) before inflating the lungs.
  **NOTE:** The volume of low-melting point agarose should be adjusted according to the size of the lungs.
2. Resect the lungs, including the distal part of the trachea, from the experimental animal.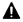 **CRITICAL STEP** Resection should be performed within 6 h after euthanasia, preferably sooner.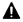 **CRITICAL STEP** Be sure not to damage the lungs while resecting, as this will interfere with the inflation process.
3. Locate the trachea (for small species) or primary bronchus (for larger species) through which you wish to inflate.
  **NOTE:** For smaller mammals, inflation through the trachea works best, but for larger animals (body weight > 3–5 kg) inflation through the primary bronchus is more efficient.
4. Fill a syringe with a 42 °C pre-warmed 1:1 mixture of 4% (*w*/*v*) low-melting point agarose and lung slice medium (DMEM/Ham’s F12 medium supplemented with 10% (*v*/*v*) FBS, penicillin, streptomycin, l-glutamine and amphotericin B). Final agarose percentage is 2% (*w*/*v*).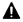 **CRITICAL STEP** Avoid the formation of air bubbles in the agarose.
  **NOTE:** Decide on the size of the syringe dependent on the size of the trachea or bronchus through which you will inflate. Either the pipette tip, blunt-end needles, catheters, cannulas the end of the syringe should be a tight fit into the trachea or bronchus.
5. Insert the end of the syringe into the trachea or primary bronchus, enforce placement inside the trachea or bronchus by clamping (or use sutures as an alternative) the trachea or bronchus around the needle.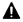 **CRITICAL STEP** Be careful not to damage the trachea or bronchus while inserting the needle into the trachea or bronchus. We advise the use of pipette tips, blunt-end needles, catheters or cannulas instead of sharp needles at the end of the syringe.
6. Inject the proper amount of agarose into the lungs. Check inflation while injecting; keep adding agarose until the lungs are completely inflated (see Video S1).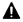 **CRITICAL STEP** It is not always easy to determine the best level of inflation. In our experience, the volume used for inflation should be slightly higher than the tidal volume of the animal, which is usually around 7 mL per kg body weight. If you are inflating just one lobe of the lung, the volume should be adapted accordingly.
7. Remove needle but keep the clamp positioned on the trachea or bronchus and allow the inflated lungs to solidify on ice (5 min for smaller species and 10–30 min for larger species).

### 3.2. Preparation of Lung Slices. Time for Completion: 1 h

1. Transfer lungs to biosafety cabinet, remove the clamp, prepare a surface for cutting (cutting board or Petri dish) and microtome blade. Prepare the lungs for slicing by finding anatomically interesting locations.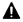 **CRITICAL STEP** Dependent on the focus of the experiment, you have to decide which part is anatomically interesting. Decide on whether you want to use for example the left or right side, and either the upper or lower parts of available lobes. To check viability, we advise to include a cut of the primary bronchus in the slice, to determine the beating of cilia indicating viable epithelial cells.
2. Start with an initial cut to ensure that a straight edge is available for cutting. Now manually prepare slices of approximately 1 mm thickness. If desired, the lungs can be attached to the cutting board by needles (see Video S2).
  **NOTE:** As a rule of thumb, slices are thin enough for culture if you can see through the slice while cutting it and actually see the microtome blade.
3. Gently transfer cut slices into 6-, 12-, 24-, or 48-well plates (dependent on size of the slices) that were pre-filled with lung slice culture medium.
4. Infection of slices can be performed immediately.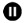 **PAUSE STEP** Slices can also be infected after 24 h of culture at 37 °C in 5% (*v*/*v*) CO_2_. We have never observed discrepancies between obtained results after either direct infection or after overnight culture.

### 3.3. Infection of Lung Slices. Time for Completion: 2 h

1. Transfer the lung slices into empty 6-, 12-, 24-, or 48-well plates (dependent on size of the slices).
2. Gently add virus (preferably a recombinant virus expressing a fluorescent reporter protein) in a drop wise fashion onto the lung slice. Typically, we used an inoculation volume of 150 μL; always include a mock infection as negative control.
  **NOTE:** We typically use 1–5 × 10^5^ Tissue Culture Infectious Dose-50 (TCID_50_) per slice; variations are possible.
3. Incubate at 37 °C in 5% (*v*/*v*) CO_2_ for 1 h.
4. After 1 h, add an appropriate volume of lung slice medium (inoculum can be washed away) and return plates to 37 °C in 5% (*v*/*v*) CO_2_.
5. In our experience, slices can be held viable (based on beating cilia in epithelium) and followed in time for a maximum of 7 days. Culture medium has to be replaced to ensure viability every other day (i.e., day 2, 4 and 6).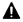 **CRITICAL STEP** When replacing culture medium, medium should be taken off and directly replaced, not allowing the lung slices to dehydrate.
  **NOTE:** Beating of cilia is the best measure for viability, however when beating of cilia is no longer observed slices start disintegrating. Depending on the goal of the experiment, non-viable slices should be removed or kept in culture.

### 3.4. Follow-Up in Time (Viability). Time for Completion: Up to 7 Days

1. Slices should be checked for viability on daily basis: find a bronchus with cilia under a normal light microscope. Movement (see Video S3) of cilia is indicative of viable epithelial cells, suggesting there are live cells in culture.

### 3.5. Follow-Up in Time (Macroscopic and Microscopic Fluorescence). Time for Completion: Up to 7 Days

**Figure 2:**
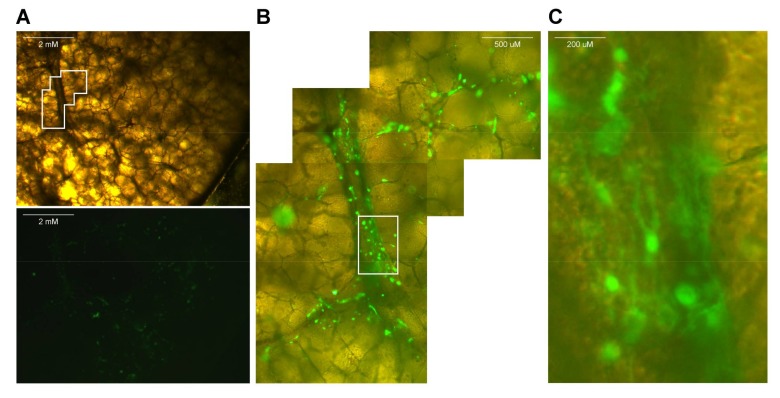
Live screening of lung slices infected with a paramyxovirus expressing a fluorescent reporter protein (green). In this example, a macaque lung slice infected with recombinant measles virus is shown. (**A**) Normal light and fluorescence image of infected lungs slice. Contrast of lower panel has been enhanced to improve visibility of the green fluorescent protein (GFP) signal. (**B**) Magnification of inset in (**A**); (**C**) Magnification of inset in (**B**).

1. If fluorescent reporter viruses were used, slices should be checked for fluorescence on a daily basis (see Video S4 and Figure 2).
2. Use a macroscopic imaging lamp as described previously [7] in combination with an emission filter to visualize fluorescence macroscopically. Shine lamp directly on lung slices and find fluorescent spots. Photographs can be acquired if desired.
3. Use an inverted UV microscope or confocal laser scanning microscope to identify fluorescent-positive cells.

### 3.6. Follow-Up in Time (Flow Cytometry). Time for Completion: Up to 7 Days

1. During infection and culture, effluent cells can be harvested and checked for expression of fluorescent proteins by flow cytometry if desired.
2. Harvest supernatants from slices (e.g., on day 2, 4 and 6 while replacing culture medium), transfer supernatant to tubes and centrifuge (5–10 min, 300× *g*).
3. Transfer pellets to 96-wells V-bottom plate, wash once in FACS buffer (PBS + 0.5% (*v*/*v*) BSA + 2 mM EDTA).
4. **OPTIONAL STEP** To determine which subsets of cells are infected, a fluorescence activated cell sorter (FACS) staining for specific cell types can be performed at this point.
  **NOTE:** Cells that migrate out of the lung slices are mainly lymphoid, myeloid or epithelial in origin. To study the tropism of infection you can selectively stain for example T-cells, B-cells, natural killer (NK) cells, neutrophils, granulocytes, dendritic cells, macrophages or epithelial cells, or combinations thereof.
5. Acquire cells on flow cytometer to determine number of virus-positive cells.

### 3.7. End of Experiment

Multiple experiments can be performed at the end of culture or at desired time points. Slices can be directly stained for confocal laser scanning microscopy (Section 3.7.1), fixed for immunohistochemistry (Section 3.7.2) or used to generate a single cell suspension for flow cytometry (Section 3.7.3).

#### 3.7.1. End of Experiment—Confocal Laser Scanning Microscopy. Time for Completion: 2 h

**Figure 3:**
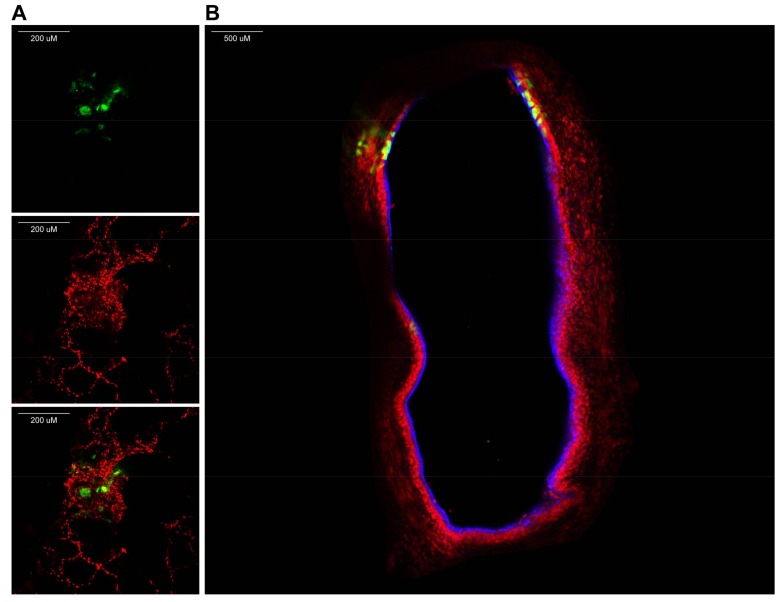
Confocal laser-scanning microscopy of lung slice infected with a paramyxovirus expressing a fluorescent reporter protein (green). In this example, a macaque lung slice infected with recombinant measles virus is shown. (**A**) Slice counterstained for nuclei (red); (**B**) Slice counterstained for nuclei (red) and cilia (blue).

1. Fix lung slices by transferring slices into new plates pre-filled with 4% (*w*/*v*) paraformaldehyde (PFA) in PBS.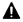 **CRITICAL STEP** Not all fluorophores are well-preserved by PFA fixation. Sensitivity of fluorophores to fixation should be tested beforehand, and the fixative of mounting medium can be adjusted accordingly.
2. Wash slices after 10 min and permeabilize in 0.1% (*v*/*v*) Triton X-100 for 30 min.
3. Wash slices and subsequently stain with fluorescent antibodies of choice.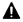 **CRITICAL STEP** To understand tissue morphology, it is important to counterstain nuclei (for example with TO-PRO-3 or NucBlue) and add a marker of choice. We found it useful to stain cilia using an antibody against class IV β-tubulin.
4. Transfer slices to thin-bottom/glass-bottom dishes.
5. View fluorescence by confocal laser scanning microscopy (using an inverted microscope, see Figure 3).
  **NOTE:** Since slices are approximately 1-mm thick, z-stacks can be generated and from those 3D images can be rendered.

#### 3.7.2. End of Experiment—Immunohistochemistry. Time for Completion: 3 Days

1. Fix lung slices by transferring slices into new plates pre-filled with 10% (*v*/*v*) formalin or directly boxing slices into cassettes kept in formalin, leave overnight and continue with paraffin embedding for immunohistochemistry.

#### 3.7.3. End of Experiment—Single Cell Flow Cytometry. Time for Completion: 3 h

1. Wash slices twice in Hank’s balanced salt solution (HBSS).
2. Transfer slices into petri dishes and manually cut in small pieces.
3. Transfer pieces into 6-well plate pre-filled with HBSS + collagenase (300 units/mL) + DNAse (0.15 mg/mL).
4. Incubate for 1 h on rocking platform at 37 °C in 5% (*v*/*v*) CO_2_.
5. Prepare single cell suspension by straining small pieces over 100 μm cell strainer.
6. Centrifuge and lyse red blood cells by adding 3 mL red blood cell lysis buffer to pellet, incubate 37 °C in 5% (*v*/*v*) CO_2_ for three minutes.
7. Centrifuge, resuspend pellet in FACS buffer and perform FACS staining of choice.

### 3.8. Ex Vivo Lung Slice Analysis of In Vivo Infected Animals

In addition to using lung slices as an ex vivo culture and infection system, the method can also be used to screen the lungs of in vivo virus-infected animals for infection and determine the viral tropism (e.g., see reference [3]). Two approaches are possible: instead of inflating lungs with the 1:1 mixture of 4% (*w*/*v*) low-melting point agarose and lung slice medium, prepare a 1:1 mixture of 4% (*w*/*v*) low-melting point agarose and 4% (*w*/*v*) PFA in PBS. The rest of the protocol is identical, after preparing lung slices these can be screened by fluorescence microscopy directly, virus-positive slices can be processed further as desired (immunohistochemistry, staining for confocal laser scanning microscopy, preparation of single cell suspensions for flow cytometry). As an alternative, slices can be inflated with 1:1 mixture of 4% (*w*/*v*) low-melting point agarose and lung slice medium and kept in culture for a period of time after necropsy, to evaluate viral tropism and dissemination [4].

## 4. Expected Results

With the protocol described here, dependent on the size of the species used in the experiment, viable lung slices from potentially any species can be obtained for ex vivo experiments. We have observed viability of these slices for up to seven days post resection; however, this could be dependent on the species and culture conditions. Infections with respiratory viruses of lung slices are normally relatively successful: Figure 2 and Figure 3 show examples of ex vivo paramyxovirus-infected lung slices. In these experiments, macaque lungs were inflated and infected with MV, which is clearly able to infect the macaque lung slices. Of course, viruses corresponding to the target species should be chosen to obtain positive results. In the family of *Paramyxoviridae* for example, viruses exclusively infecting primates or carnivores exist. Measles virus, a virus of primates, successfully infected non-human primate but not dog lungs, whereas CDV, a virus of carnivores, behaved vice versa. Using viruses expressing fluorescent reporter proteins allows for sensitive detection of virus-infected cells in these experiments; however, these experiments can also be performed with non-fluorescent viruses. Staining should still make infected cells visible in immunohistochemistry, confocal laser scanning microscopy or flow cytometry.

The model described here of course has some limitations, as a single region of the lung is directly infected, often with a relatively high inoculum. Therefore, in vivo experiments in animal models of disease are of crucial importance for viral tropism and pathogenesis studies. However, these experiments must be complemented with proper in vitro and ex vivo experiments, indicating the potential of ex vivo experiments in these cultured lung slices.

## 5. Reagents Setup

- 4% agarose: prepare correct weight of hydroxyethylagarose in DPBS;
- Lung slice medium: DMEM/Ham’s Nutrient Mixture F12 powder, Glutamax, FBS, Penicillin/Streptomycin, amphotericin;
- Fluorescence activated cell sorter buffer: PBS + 0.5% (*v*/*v*) BSA + 2 mM EDTA (use stock of 0.5 M in distilled water);
- Paraformaldehyde: dissolve appropriate weight in PBS with high pH by initially supplementing with NaOH. After PFA has completely dissolved, pH should be re-adjusted to 7.4. Always prepare fresh or store aliquoted at −20 °C. Do not use buffered formalin when directly screening fluorescence, as this will disrupt the fluorescence of most reporter proteins.

## Supplementary Materials

The following are available online at http://www.mdpi.com/2409-9279/1/2/12/s1. Video S1: Inflation of cotton rat lungs. Syringe is filled with agarose mixed with lung slice medium and inserted into the trachea, kept in place by a suture. Lungs are slowly inflated by injecting agarose and medium into the trachea; Video S2: Slicing of cotton rat lungs. Solidified cotton rat lungs are places on petri-dish and subsequently sliced. An initial cut is made for a straight edge followed by the preparation of a single lung slice; Video S3: Viability screening of lung slice. A bronchus consisting of viable epithelial cells with beating cilia; Video S4: Fluorescence screening of live lung slice infected with a paramyxovirus expressing a fluorescent reporter protein (green). The movie initially shows a combination of normal light and fluorescent light, followed by a shutdown of the normal light, indicating the identical area but now with fluorescence light only.
